# Consensus on the pharmacological treatment of acute stress disorder in Chinese pilots: a Delphi study

**DOI:** 10.1186/s12888-023-05145-5

**Published:** 2023-09-08

**Authors:** Xing Gao, Zhenzhen Wang, Li Guo, Yanan Gu, Lei Song, Zhongying Wu, Fengzhan Li, Yinchuan Jin, Qun Yang

**Affiliations:** 1 Department of Military Medical Psychology, The Fourth Military Medical University, Xi’an, 710032 China; 2 Air Force Hospital of Southern Theater Command, Guangzhou, 510000 China; 3 The Fourth Military Medical University, Xijing hospital, Xi’an, 710032 China

**Keywords:** Pilots, Acute stress disorder, Drug treatment, Delphi method

## Abstract

**Background:**

Appropriate medication is very important for pilots with acute stress disorder. Improper medication can not only affect the physical and mental health of the pilots but can also endanger flight safety. Hence, we aimed to quickly and effectively relieve symptoms and restore cognitive function by forming a consensus of Chinese experts on the pharmacological treatment of acute stress disorder in pilots using the Delphi method.

**Methods:**

Relevant literature was searched to enumerate the current status of pharmacological treatment of acute stress disorder in pilots, followed by two rounds of expert consultation and discussion according to the listed status of the survey using the Delphi method. A descriptive statistical method was used to analyze the basic information, authority coefficients, concentration of opinions, and survey items of the experts to develop a consensus on the pharmacological treatment of acute stress disorder in pilots.

**Results:**

A total of 16 experts in psychiatry, pharmacology, and aerospace medicine from different provinces and cities across China were invited for consultation. The recovery rate of the two rounds of consultation was 100%, and the expert authority coefficients were 0.897 and 0.906, respectively. Kendall’s coefficient of concordance of indicators at all levels was 0.564–0.594 (p < 0.01). Based on the number of votes received, alprazolam tablets (16), eszopiclone tablets (15), and lorazepam tablets (14) were recommended for the treatment of excitatory psychomotor symptoms of acute stress disorder; paroxetine tablets (15) and sertraline tablets (15) were available for psychomotor depressive symptoms; olanzapine tablets (15), olanzapine orally disintegrating tablets (14), and quetiapine fumarate tablets (14) were selected for psychotic symptoms.

**Conclusions:**

This study formed a consensus on rapid and effective pharmacological treatment for different symptoms of acute stress disorder pilots, which provides a reference for clinical treatment.

**Supplementary Information:**

The online version contains supplementary material available at 10.1186/s12888-023-05145-5.

## Background

Based on strictly medical and psychological criteria, pilots showed higher physical and psychological fitness than the general population. Also, flight stress and special occupational environments are different from the general population, such as long-term continuous observation and control, excessive noise and vibration, and machine failure, which make them prone to produce acute stress disorder (ASD) [1, 2]. Specifically, the outbreak of coronavirus disease-2019 (COVID-19) increased psychological pressure, anxiety, depression, and insomnia, further aggravating ASD [3]. ASD is a transient physiological and psychological reaction that occurs immediately after an individual suddenly encounters a mental trauma [4]. It is mainly manifested as psychomotor excitement with an intense fear experience and is presented as blindness or psychomotor inhibition, numbness, or psychotic symptoms. It lasts for three days to one month [5]. Without timely intervention, 20–50% of patients with ASD are transformed into posttraumatic stress disorder(PTSD), causing greater psychological and physical damage [6]. As the direct operator of aircraft, the pilots’ mental health level is crucial for flight safety. Once ASD occurs, the pilots’ cognitive function, perception, and attention will be reduced [7], which will lead to adverse emotions, such as irritability and anxiety, as well as physiological reactions, such as increased heart rate, blood pressure, and cerebral blood flow, further leading to operational errors and affecting flight safety [8–10]. Therefore, the timely and effective intervention in treatment of pilots with ASD is one of the key issues for clinical attention.

When pilots with ASD stop flying missions for medical attention, the treatment plan adopted is the same or similar to that of the general population. The treatment of ASD can be divided into three main types: pharmacotherapy, psychotherapy, and physical therapy. Although psychotherapy, such as eye movement desensitization reprocessing therapy and trauma-focused cognitive behavioral therapy, can improve the patients’ negative emotions and help them rebuild cognition [6]. Therapists are required to provide treatment; nonetheless, the effect is slow and costly, and the treatment effect varies individually in patients. Physical therapy, such as transcranial direct current stimulation and transcutaneous cervical vagal nerve stimulation, is reported in several cases, but no systematic studies have focused on different treatment methods and therapeutic effects. Conversely, the cost of drug treatment is low, and its short-term use can rapidly control the symptoms of patients, prevent excessive behavioral changes, and achieve satisfactory therapeutic effects [11, 12].Therefore, pharmacological treatment was the main focus of this study.

However, currently, there is no consensus on the drug treatment of ASD; sertraline, paroxetine, fluoxetine, and venlafaxine, are administered as recommended by Australian guidelines [13]. The Chinese guidelines recommend imipramine, fluoxetine, risperidone, and propranolol [14]. Simultaneously, most of the guidelines do not recommend drugs for various specific symptoms of ASD. As mentioned earlier, ASD manifests as psychomotor arousal and other symptoms, and different drugs can effectively treat various symptoms [15]. In addition, only a few clinical studies reported differences in the drug treatment schemes among various regions and hospitals, such as paroxetine and citalopram for ASD in a hospital in Xinjiang, China [16], olanzapine for ASD in a hospital in Jiangsu, China [11], prazosin for ASD in a hospital in New Jersey, USA [17], and fluoxetine and imipramine for ASD in a hospital in Texas, USA [18]. Also, only a few studies have focused on pilots, who form a special group. Nonetheless, a large number of studies have shown that psychotropic drugs may damage attention, cause drowsiness, and extrapyramidal adverse effects, which otherwise cure ASD in the pilots, but may affect their flight operating ability due to adverse drug effects. Therefore, the present study was aimed to select drugs that can quickly and effectively relieve the symptoms and restore cognitive function.

For this study many experts in clinical psychiatry, pharmacology, and aerospace medicine were invited to reach a consensus on the pharmacological treatment of ASD symptoms in pilots by the Delphi method in order to rapidly and effectively relieve symptoms and restore cognitive function through medications only. This research initiative is for an emergency treatment for pilots with unexpected symptoms of ASD and does not include pilots who are receiving psychiatric medication.

## Methods

### Study design

Delphi method can collect information from expert groups to reach a consensus on related problems [19] and is increasingly used to form a consensus on the treatment of mental and psychological problems [20]. The current Delphi study involved four stages:(1) establishment of a study group, (2) questionnaire development, (3) identification and recruitment panels, and (4) expert consultation.

### Establishment of a study group

A 7-member research group was established with three clinical psychologists, one psychiatrist, one statistician, and two postgraduates in clinical psychology. The average age of the clinical psychologists was 41 ± 8-years-old. The task of the research team was to compile the consultation questionnaire, establish consulting experts, seek advice from the experts on Delphi method, conduct statistical analysis, and assimilate the experts’ opinions.

### Questionnaire development

The research team searched PubMed, CNKI (China National Knowledge Infrastructure), and Web of Science databases from their inception to June 2021 for systematic literature retrieval. The keywords used were: pilots, stress disorders, traumatic, acute, acute stress reaction, drug treatment. A total of 17 commonly used drugs for ASDand their adverse effects were listed for ASD treatment.

The questionnaire consisted of three parts: description, basic information of experts, and a text. (1) The questionnaire description included the research background, purpose, and significance; (2) Basic information of experts included gender, age, educational background, professional title, working years, research field, the educational level of the expert and the basis for judgment V(Ca)(Additional file 1), and the degree of the expert’s familiarity with the indicators V(Cs) (Additional file 2); (3) The main body of the questionnaire included whether it can be used for ASD pilot patients, symptoms of ASD (psychomotor excitement, psychomotor inhibition, and psychotic symptoms), and feasibility of use and popularization. The responses were judged using a Likert 5-point scale: from 1 point (unimportant) to 5 points (very important), and comments and on suggestions given as additional items by the experts.

### Identification and recruitment panels

The sampling method, including 16 experts from pharmacology, psychiatry, and aerospace medicine, across top hospitals in Beijing, Guangzhou, Dalian, Chongqing, and other provinces and cities in China, were invited to form a consulting expert group (Additional file 3). The inclusion criteria of the expert group was age > 35-years-old; intermediate title or above; engaged in this profession for > 10 years; informed consent and active participation in this study.

The role of the expert group was to put forward opinions and suggestions on the content of consultation, to rate each project, and to remain anonymous in the process. The participation was voluntary, informed consent was obtained, and each person who completed all the consultations received a 1000 RMB (an official currency of China) award for their consultation services according to the project fund document’s standard. The research team formed a consensus on pilots’ ASD drug treatment according to experts’ suggestions. Therefore, the opinions and recommendations of the expert group determine the authority of the consensus.

### Process of consultation with experts

**Fig. 1:**
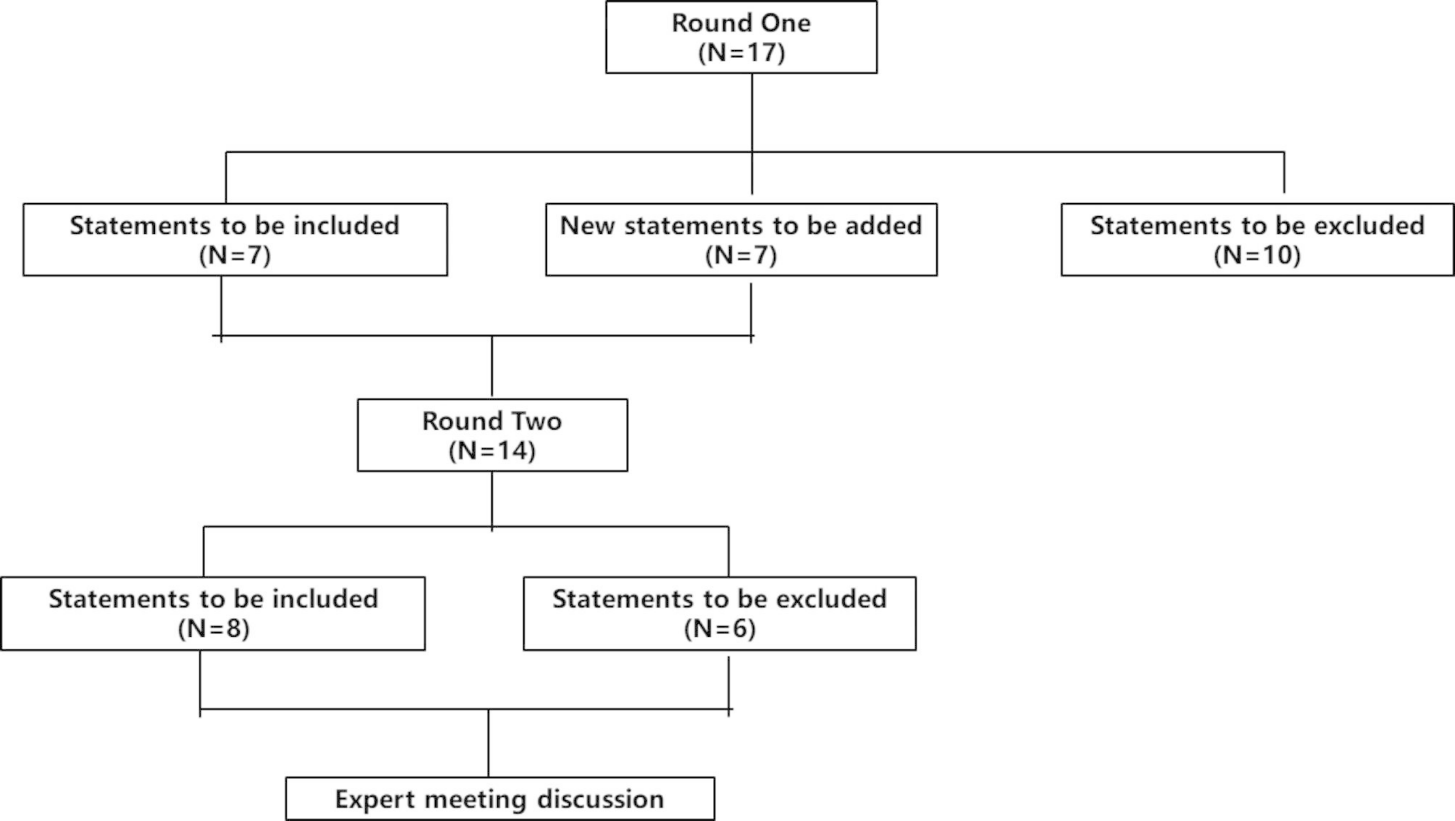
The whole process of the study on ASD treatment to air pilots

The study was conducted by distributing questionnaires on site or by email. The results of the first round of investigation were analyzed, and the responses to the second round of questionnaires were compiled. The results of the first round were attached to the second round of questionnaires for investigation. After two rounds of investigation, an experts’ meeting was held to discuss and reach a consensus. Figure 1 illustrates the whole process of this study.

SPSS version 19.0 software was used to analyze the data. The degree of expert authority was expressed by the authority coefficient (Cr), which was evaluated by the expert’s familiarity with indicators V(Cs) and the expert’s judgment V(Ca). In the case of V(Cs), score 1 point is for more familiar, 0.8 points for familiar, 0.6 points for general, 0.4 points for unfamiliar, and 0.2 points for ignorant. The value of V(Ca) is 0.3, 0.2, and 0.1 for theoretical analysis, 0.5, 0.4, 0.1 for practical experience, and 0.1 for reference materials and intuition. The formula is Cr=(Cs + Ca)/2. The feasibility and popularization were scored according to the Likert 5-point scale, with scores 1 representing poor feasibility and popularization and 5 representing good feasibility and popularization. The concentration degree of experts’ opinions was expressed by the percentage, average value, and standard deviation of the project agreement. The coefficient of variation (CV) was used to evaluate the consistency of experts’ opinions on the indicators. In addition, we used Kendall’s W to test the consistency of the experts’ opinions. In this study, a consensus was defined as the proportion of pilot patients who agreed to be subjects for ASD > 80%; the mean value of feasibility and popularization ($\bar x$) > 3.5, and the CV(%) <25 must be in agreement before inclusion.

## Results

### Expert panel information

The baseline demographics experts are shown in Table 1. A total of 16 experts in psychiatry, pharmacology, and aerospace medicine from 8 different provinces and cities in China, such as Beijing and Guangzhou, were invited for consultation. Among them, 13 experts completed all the consultations, while three experts only participated in the first round of consultation and replaced those with the same specialty and similar qualifications in the second round of consultation and subsequent expert meetings. The age of the experts was between 40- and 60-years-old, 75% of them had a graduate degree, 93.75% had senior professional titles, and 75.5% had been engaged in this major for > 20 years.

**Table 1:** Basic demographic data of experts

Demographics	First round N (%)	Second round N (%)
Gender		
male	8(50)	10(63)
female	8(50)	6(38)
Age		
30 to 39 years old	1(6)	1(6)
40 to 49 years old	5(31)	6(38)
50 to 59 years old	8(50)	7(44)
≥ 60 years old	2(13)	2(13)
Academic Degree		
undergraduate	4(25)	4(25)
master	6(38)	8(50)
doctor	6(38)	4(25)
Professional Title		
intermediate title	1(6)	1(6)
senior title	15(94)	15(94)
Working Life		
8 to 20 years	4(26)	4(26)
21 to 30 years	8(50)	8(50)
over 31 years	4(26)	4(26)
Research Field		
psychiatry	14(88)	14(88)
aerospace medicine	1(6)	1(6)
pharmacology	1(6)	1(6)

N (%):Total number (Percentage of the total number)

### Effectiveness of delphi method

The recovery rate of the two rounds of expert consultation forms in this study was 100%. In the two rounds of consultations, the expert authority coefficients were 0.90 and 0.91, respectively. The feasibility and popularization of Kendall’s W of the first round were 0.591 (P < 0.01) and 0.564 (P < 0.01), respectively, while those of the second round were 0.594 (P < 0.01) and 0.575 (P < 0.01), respectively (Table 2).

**Table 2:** Concordance coefficients of respondent experts

Hierarchical level	First round				Second round				
	N	Kendall’s W	χ^2^	P	N	Kendall’s W	χ^2^	P	
Feasibility	16	0.591	37.822	0.002	16	0.594	92.699	< 0.001	
Popularization	16	0.564	36.127	0.003	16	0.575	89.685		< 0.001

Kendall’s W: Kendall’s coefficient of concordance. χ^2^: chi-square test. P: Kendall’s w, significance was set a *P* < 0.05

### Expert statement

Based on consent for ASD > 80%, mean of feasibility and prevalence(‾x)> 3.5 and CV(%) <25, all the above conditions were met for inclusion. According to the results of the first round of expert consultation, seven items were reserved, including alprazolam tablets, paroxetine tablets, sertraline tablets, olanzapine tablets, lorazepam tablets, venlafaxine tablets, and propranolol tablets, while tenothers (haloperidol injection, clonazepam tablets, hydrocortisone tablets, imipramine tablets, citalopram tablets, diazepam injection, risperidone tablets, estazolam tablets, fluoxetine tablets, and diazepam tablets) were deleted (Table 3). According to experts’ suggestions, seven additional items were added for the second round of consultation, including olanzapine orally disintegrating tablets, risperidone oral liquid, quetiapine fumarate tablets, estazolam injection, flupentixol melitracen tablets, zopiclone tablets, and eszopiclone tablets.

The inclusion criteria of the second round were the same as those of the first round, and eight items namely, alprazolam tablets, lorazepam tablets, paroxetine tablets, sertraline tablets, olanzapine tablets, olanzapine orally disintegrating tablets, quetiapine fumarate tablets, and eszopiclone tablets, were reserved (Table 4).

Finally, an expert meeting was held, and experts (n = 16) unanimously agreed on the consultation results and reached a consensus. The specialists classified the medications based on their professional knowledge. According to the voting results, alprazolam tablets (16), eszopiclone tablets (15), and lorazepam tablets (14) were selected for the psychomotor excitement symptoms in ASD; paroxetine tablets (15) and sertraline tablets (15) were used for the symptoms of psychomotor inhibition; olanzapine tablets (15), olanzapine orally disintegrating tablets (14), and quetiapine fumarate tablets (14) were selected for psychotic symptoms (Table 5).

**Table 3:** Results of the first round of expert consultation

Medicine	Class	Agreed to be used (%)	Feasibility		Popularization	
			Mean (SD)	CV(%)	Mean (SD)	CV(%)
Diazepam Tablets	Benzodiazepine	93.75	3.33(0.79)	24	3.20(0.65)	20
Inj Diazepam	Benzodiazepine	81.25	3.62(0.84)	23	3.46(0.93)	27
Clonazepam tablets	Benzodiazepine	75	3.25(1.23)	38	2.92(1.26)	43
Alprazolam tablets	Benzodiazepine	87.5	3.71(0.45)	12	3.64(0.89)	25
lorazepam tablets	Benzodiazepine	81.25	3.92(0.83)	21	3.77(0.80)	21
Estazolam tablets	Benzodiazepine	93.75	3.53(0.96)	27	3.47(0.88)	26
Imipramine tablets	Tricyclic antidepressant	43.75	2.43(0.49)	20	2.14(0.35)	16
Paroxetine tablets	Selective serotonin reuptake inhibitor	93.75	3.80(0.54)	14	3.80(0.65)	17
Sertraline tablets	Selective serotonin reuptake inhibitor	87.5	3.86(0.64)	17	3.79(0.67)	18
Citalopram tablets	Selective serotonin reuptake inhibitor	81.25	3.85(1.03)	27	3.85(1.10)	29
Fluoxetine tablets	Selective serotonin reuptake inhibitor	75	3.83(0.69)	18	3.67(0.62)	17
Venlafaxine tablets	Serotonin norepinephrine reuptake inhibitor	81.25	3.77(0.70)	18	3.54(0.84)	24
Risperidone tablets	Second-generation antipsychotic	81.25	3.62(0.84)	23	3.62(1.00)	28
Olanzapine Tablets	Second-generation antipsychotic	93.75	4.27(0.57)	13	4.20(0.83)	20
Haloperidol injection	First-generation antipsychotic	81.25	3.85(0.77)	20	3.62(0.92)	26
Propranolol tablets	Beta blockers	81.25	3.77(0.58)	15	3.77(0.58)	15
Hydrocortisone tablets	Glucocorticoid	50	2.63(0.99)	38	2.63(0.99)	38

SD: Standard Deviation, CV(%): coefficient of variation

**Table 4:** Results of the second round of expert consultation

Medicine	Class	Agreed to be used (%)	Feasibility		Popularization	
			Mean (SD)	CV(%)	Mean (SD)	CV(%)
Alprazolam tablets	Benzodiazepine	100	4.56(0.50)	11	4.50(0.50)	11
Paroxetine tablets	Selective serotonin reuptake inhibitor	100	4.63(0.48)	10	4.69(0.46)	10
Sertraline tablets	Selective serotonin reuptake inhibitor	100	4.44(0.50)	11	4.44(0.50)	11
Olanzapine Tablets	Second-generation antipsychotic	93.75	4.67(0.47)	10	4.53(0.50)	11
lorazepam tablets	Benzodiazepine	93.75	3.93(0.57)	15	3.73(0.57)	15
Venlafaxine tablets	Serotonin norepinephrine reuptake inhibitor	100	3.44(0.70)	20	3.56(0.70)	20
Propranolol tablets	Beta blockers	93.75	3.00(0.73)	24	2.93(0.68)	23
Flupentixol and Melitrace tablets	Antidepressant	81.25	3.15(0.66)	21	3.08(0.62)	20
Olanzapine orally disintegrating tablets	Second-generation antipsychotic	93.75	4.53(0.50)	11	4.60(0.49)	11
Quetiapine Fumarate Tablets	Second-generation antipsychotic	93.75	3.67(0.47)	13	3.53(0.50)	14
Zopiclone tablets	Cyclopyrrolones	87.5	3.14(0.64)	20	3.21(0.67)	21
Eszopiclone tablets	Cyclopyrrolones	93.75	3.60(0.80)	22	3.60(0.80)	22
Risperidone oral liquid	Second-generation antipsychotic	100	3.63(0.60)	17	3.50(0.61)	17
Estazolam injection	Benzodiazepine	93.75	3.07(0.85)	28	3.00(0.82)	27

SD: Standard Deviation, CV(%): coefficient of variation

**Table 5:** Agreed for each drug results for various symptoms

Project	Eszopiclone tablets	Alprazolam tablets	lorazepam tablets	Paroxetine tablets	Sertraline tablets	Olanzapine Tablets	Olanzapine orally disintegrating tablets	Quetiapine Fumarate Tablets
Excitement symptoms	15	16	14	2	1	7	6	7
Suppress symptoms	3	0	2	15	15	3	2	1
Psychotic symptoms	3	4	3	2	1	15	14	14

The number in the table represents the number of people who endorsed each symptom for each drug

## Discussion

The present study aimed to identify medications that may rapidly and effectively relieve the ASD symptoms and recover the cognitive function in air pilots; 16 experts in clinical psychiatry, pharmacology, and aerospace medicine were invited to reach a consensus on the pharmacological treatment of pilots’ ASD symptoms by the Delphi method to provide a reference for the clinical pharmacological treatment. The recovery rates of the two rounds of expert consultations in this study were 100%, and the expert authority coefficients were 0.90 and 0.91, respectively, indicating that the invited experts were very concerned about this study and had some authoritative representatives in this field confirming the reliability [21]. The experts’ scores on the agreed for use in air pilots suffering ASD reflect their recognition of the item indicating a higher score, for a higher level of endorsement. Therefore, item with > 80% was chosen as one of the inclusion indexes. The mean value reflected on the degree of concentration of experts’ opinions, and the CV indicated the consistency of experts’ opinions. In addition, the average score of feasibility and popularization > 3.5 and the CV(%) < 25 served as the inclusion criteria. Kendall’s W is a commonly used index to test the consistency of experts’ opinions. In the second round, Kendall’s W was 0.594 (P < 0.01) and 0.575 (P < 0.01), respectively, indicating expert consultation can be closed [22]. The expert consultation voting process are uploaded in Additional file 4.

In the present study, clonazepam, diazepam, and estazolam were first excluded by aerospace medicine and psychiatry experts, because clonazepam and diazepam can damage the memory and cause depression [23]. Estazolam has a long half-life, which easily leads to drowsiness, lethargy, and other adverse reactions and irritability, anxiety, and other symptoms after withdrawal from the drug [24]. Almost all the experts in aerospace medicine, pharmacology, and psychiatry agreed on the use of alprazolam and lorazepam because they can significantly reduce the severity of panic and anxiety [25, 26]. Alprazolam is a high-potency triazolobenzodiazepine that has been licensed by the United States Food and Drug Administration (FDA) for the treatment of anxiety and panic disorders. It decreases circulating corticosterone levels dramatically in the early days of acute trauma, significantly attenuates the hypothalamic-pituitary-adrenal axis (HPA axis) response, substantially reduces anxiety [27] and can be given to pregnant women [28]. Lorazepam is effective in relieving anxiety in the acute attack [29]and is used in the elderly because of its high safety profile [30]. Psychiatry experts suggested adding the use of eszopiclone. This recommendation may be attributed to the fact that REM sleep plays a positive role in the emotional integration of painful memory, and improving patients’ sleep to effectively relieve ASD symptoms [31, 32]. With its high biological activity, sedation, hypnotic effects, and fewer adverse reactions, Almost all experts od aerospace medicine, pharmacology, and psychiatry agreed that eszopiclone is suitable for an effective treatment of ASD pilots [33].

Reportedly, imipramine, sertraline, citalopram, and venlafaxine are often used to treat ASD patients with psychomotor depression symptoms [34, 35]. Aerospace medicine specialist and half of the psychiatrists opposed the use of imipramine. They pointed out that imipramine relieved the symptoms, such as insomnia, depression, nightmares, and flashbacks of ASD patients, but tended to cause mania [36]. Some psychiatrists opposed the use of fluoxetine, citalopram, venlafaxine, flupentixol and melitrace. Fluoxetine and citalopram do not improve cognitive function, such as attention [37]. Venlafaxine can cause dose-dependent hypertension, hypoglycemia, and other adverse reactions, and its efficacy is similar to that of selective serotonin reuptake inhibitors (SSRI) [38]. Flupentixol and melitrace stimulate the receptor of reticular ascending activating system a_1_ by increasing NE, which leads to insomnia and mania that are not favorable to the treatment of ASD; thus, were eliminated by experts in this study [33]. However, the only FDA approved treatments for trauma are two SSRIs, sertraline and paroxetine, which are effective in reducing symptoms of trauma and have an overall positive safety [39, 40]. Several clinical trials have revealed that paroxetine might prevent PTSD and associated sensations of numbness and avoidance while also being safe and tolerable [41]. Sertraline has fewer adverse effects, lowers arousal, avoidance, and numbness, as well as has a high safety profile for use in children [40]. Experts unanimously agreed that they could be used in air pilots.Propranolol and hydrocortisone are used as old drugs for new purposes, although some studies have shown that propranolol can reduce patients’ memory of stimulating emotional material and has a tendency to limit the follow-up fear-conditioned reflex [42, 43]. Hydrocortisone promote the return to steady-state in the hypothalamic-pituitary-adrenal axis (HPA axis) and damage the consolidation and recall of traumatic memory [44]; however, almost all experts in aerospace medicine and psychiatry have pointed out on the reported variability in the efficacy in a few existing studies, and hence, were not recommended.

Studies with olanzapine, quetiapine fumarate, risperidone, and haloperidol have reported antipsychotic effects in patients with ASD [11]. Nonetheless, some psychiatry experts pointed out that haloperidol and risperidone have stronger anti-dopamine D_2_ receptor effects than olanzapine and quetiapine fumarate, i.e., they are more likely to cause acute extrapyramidal reactions, tardive dyskinesia, and hyperprolactinemia. In addition, risperidone blocks the D_1_ receptor on the postsynaptic membrane of the midbrain cortical pathway, which might cause inattention and memory loss [45]. Olanzapine enhances cognitive function more effectively than risperidone and haloperidol [46]as well as reduces irritability, aggression, and insomnia [47–49]. Because it is safe and effective it is also used on children [50]. Furthermore, quetiapine has been shown to reduce flashbacks and hyperarousal symptoms, as well as trauma-related anxiety and depression [51], with the added benefit of being well tolerated and having few adverse effects [52]. Therefore, almost all experts of aerospace medicine, pharmacology, and psychiatry have recommended the use of olanzapine and quetiapine for treatment of air pilots with ASD.

### Limitations and strengths

The study has several limitations. Firstly, our study only invited Chinese specialists in aerospace medicine, pharmacology and clinical psychiatry. The key component of the work was to identify medications that may rapidly and effectively relieve the ASD symptoms and recover the cognitive function for Chinese pilots. Therefore, it applies predominantly ASD in Chinese pilots. Its applicability to the pilots of ASD in other counties may be limited. Second, due to the difficulty of contacting a limited number of such experts available, only 16 experts were invited to participate. But there is presently no agreement on the appropriate sample size for an expert panel, with some writers advocating as few as fifteen, ten, or even seven people [53, 54]. When assessing the level of representativeness of the findings from a Delphi survey, the quality of the expert panel appears to be more significant than the quantity [55]. Third, Delphi techniques are considered to provide the lowest level of evidence for making causal inferences. Although this study’s findings have a low degree of proof, they nonetheless have some practical significance. Compared to the existing guidelines, the present study not only screened out the therapeutic drugs for ASD pilots but also sorted the drugs according to the psychomotor excitement symptoms, psychomotor inhibition symptoms, and psychotic symptoms.

## Conclusion

By the Delphi method we obtained the consensus of rapid and effective identification of drugs to treat different symptoms of ASD in the pilots Alprazolam, lorazepam, and eszopiclone tablets are the recommended drugs for the treatment of psychomotor excitement symptoms of ASD. Paroxetine and sertraline tablets are the recommended drugs for the treatment of psychomotor inhibition symptoms of ASD. Olanzapine, olanzapine orally disintegrating, and quetiapine fumarate tablets are recommended for the treatment of psychotic symptoms of ASD.

### Electronic supplementary material

Below is the link to the electronic supplementary material.

Supplementary file 1: ST1. Quantitative table of the basis for expert judgement

Supplementary file 2: ST2. Expert familiarity self-assessment form

Supplementary file 3: ST3. List of experts and address

Supplementary file 4: ST4. Round1: Partial results of expert voting; ST5. Round2: Partial results of expert voting

## Data Availability

The quantitative dataset supporting the conclusions of this article are included within the article. The qualitative dataset used and analyzed during the current study are available from the corresponding author on reasonable request.

## Abbreviations

ASD: Acute stress disorder
COVID-19: Coronavirus disease-2019
PTSD: Posttraumatic stress disorder
CV: Coefficient of variation, Kendall’s W:Kendall’s coefficient of concordance
CNKI: China National Knowledge Infrastructure
FDA: The United States Food and Drug Administration
SSRIs: Selective serotonin reuptake inhibitors
HPA axis: Hypothalamic-pituitary-adrenal axis
