# Supplementary material for: Consensus on the pharmacological treatment of acute stress disorder in Chinese pilots: a Delphi study
Source: BMC Psychiatry. 2023 Sep 8;23:664. doi: 10.1186/s12888-023-05145-5 (PMC10492406; doi:10.1186/s12888-023-05145-5)
Supplement: Supplementary file 1 — ST1. Quantitative table of the basis for expert judgement [file 12888_2023_5145_MOESM1_ESM.docx]

ST1. Quantitative table of the basis for expert judgement

| Basis of expert judgement | Quantification values | | |
| --- | --- | --- | --- |
|  | Big | Medium | Small |
| Theoretical analysis | 0.3 | 0.2 | 0.1 |
| Practical experience | 0.5 | 0.4 | 0.1 |
| Reference | 0.1 | 0.1 | 0.1 |
| Intuition | 0.1 | 0.1 | 0.1 |
