# Supplementary material for: Consensus on the pharmacological treatment of acute stress disorder in Chinese pilots: a Delphi study
Source: BMC Psychiatry. 2023 Sep 8;23:664. doi: 10.1186/s12888-023-05145-5 (PMC10492406; doi:10.1186/s12888-023-05145-5)
Supplement: Supplementary file 2 — ST2. Expert familiarity self-assessment form [file 12888_2023_5145_MOESM2_ESM.docx]

ST3. List of experts and address

| Name | Address |
| --- | --- |
| Min Li | No. 30 Yanzheng Street, Gaotan, Shapingba District, Chongqing |
| Wang Wang | No. 801 Dongfeng East Road, Yuexiu District, Guangzhou |
| Dongyao Wang | No. 24, Lenin Street, Lvshunkou District, Dalian |
| Hong Yuan | No. 69 Yongding Road, Yongding Road Street, Haidian District, Beijing |
| Ronghuan jiang | No. 28 Fuxing Road, Haidian District, Beijing |
| Xiaobing Liu | No. 52 Zhizhi Road, Qingxiu District, Nanning City |
| Hongzheng Li | No. 52 Zhizhi Road, Qingxiu District, Nanning City |
| Feng Zou | No. 80, Shengli Road, Xigang District, Dalian City, Liaoning Province |
| Liqiong Zhao | No. 52 Zhizhi Road, Qingxiu District, Nanning City |
| Dongmei Yi | No. 80, Shengli Road, Xigang District, Dalian City, Liaoning Province |
| Tao Xu | No. 15 Yanggongdi, Xihu District, Hangzhou |
| Jie Shi | No. 16, Xinjiekouwai Street, Xicheng District, Beijing |
| Zhongyuan Yu | No. 111, Liuhua Road, Yuexiu District, Guangzhou City, Guangdong Province |
| Zhiguo Sun | No. 52 Zhizhi Road, Qingxiu District, Nanning City |
| Xiuzhen Li | No. 116, Zaojiatun Village, Shangzhuang Town, Haidian District, Beijing |
| Tianchao Xu | No. 83, Wenhua Road, Shenhe District, Shenyang |
| Jianquan Tian | No. 338 Huaihai West Road, Changning District, Shanghai |
| Min Song | No. 10 Huifeng Road, Liangxi District, Wuxi City |
