# Supplementary material for: Consensus on the pharmacological treatment of acute stress disorder in Chinese pilots: a Delphi study
Source: BMC Psychiatry. 2023 Sep 8;23:664. doi: 10.1186/s12888-023-05145-5 (PMC10492406; doi:10.1186/s12888-023-05145-5)
Supplement: Supplementary file 3 — ST3. List of experts and address [file 12888_2023_5145_MOESM3_ESM.docx]

We displayed a portion of the voting procedure, number 11 was an aerospace medical expert, number 16 was a pharmacologist, and the rest were clinical psychiatrists. ST4 and ST5 used a Likert 5 rating scale of 1 (very poor)-5 (very good), with the number 0 indicating that the experts did not think it was acceptable for ASD pilots. ST6 revealed some of the results of the third round of expert voting on the application of different medications to various symptoms.

ST4. Round1: Partial results of expert voting

| Expert Number | Alprazolam tablets  Feasibility Popularization | | lorazepam tablets  Feasibility Popularization | | Paroxetine tablets  Feasibility Popularization | | Sertraline tablets  Feasibility Popularization | | Olanzapine Tablets  Feasibility Popularization | | | Hydrocortisone tablets  Feasibility Popularization | | |
| --- | --- | --- | --- | --- | --- | --- | --- | --- | --- | --- | --- | --- | --- | --- |
| 1 | 3 | 2 | 3 | 3 | 3 | 3 | 3 | 3 | | 0 | 0 | | 0 | 0 |
| 2 | 0 | 0 | 0 | 0 | 4 | 5 | 5 | 5 | | 4 | 4 | | 4 | 4 |
| 3 | 4 | 5 | 0 | 0 | 3 | 3 | 4 | 4 | | 5 | 5 | | 3 | 3 |
| 4 | 4 | 4 | 5 | 5 | 4 | 4 | 0 | 0 | | 4 | 4 | | 0 | 0 |
| 5 | 4 | 4 | 4 | 4 | 0 | 0 | 0 | 0 | | 3 | 3 | | 0 | 0 |
| 6 | 3 | 2 | 5 | 4 | 4 | 4 | 3 | 3 | | 5 | 5 | | 0 | 0 |
| 7 | 4 | 4 | 3 | 3 | 4 | 3 | 4 | 4 | | 4 | 3 | | 1 | 1 |
| 8 | 4 | 4 | 3 | 3 | 3 | 3 | 3 | 3 | | 4 | 3 | | 2 | 2 |
| 9 | 4 | 3 | 4 | 4 | 4 | 4 | 4 | 4 | | 5 | 5 | | 0 | 0 |
| 10 | 4 | 4 | 3 | 3 | 3 | 3 | 3 | 3 | | 4 | 3 | | 2 | 2 |
| 11 | 4 | 5 | 4 | 3 | 4 | 4 | 4 | 4 | | 4 | 5 | | 0 | 0 |
| 12 | 4 | 4 | 5 | 5 | 4 | 4 | 4 | 4 | | 5 | 5 | | 3 | 3 |
| 13 | 4 | 4 | 0 | 0 | 4 | 4 | 4 | 4 | | 4 | 4 | | 0 | 0 |
| 14 | 3 | 3 | 5 | 5 | 4 | 4 | 4 | 3 | | 4 | 5 | | 2 | 2 |
| 15 | 3 | 3 | 4 | 4 | 5 | 5 | 5 | 5 | | 5 | 5 | | 0 | 0 |
| 16 | 0 | 0 | 3 | 3 | 4 | 4 | 4 | 4 | | 4 | 4 | | 4 | 4 |

ST5. Round2: Partial results of expert voting

| Expert Number | Alprazolam tablets  Feasibility Popularization | | lorazepam tablets  Feasibility Popularization | | Paroxetine tablets  Feasibility Popularization | | Sertraline tablets  Feasibility Popularization | | Olanzapine Tablets  Feasibility Popularization | | | Eszopiclone tablets  Feasibility Popularization | | |
| --- | --- | --- | --- | --- | --- | --- | --- | --- | --- | --- | --- | --- | --- | --- |
| 1 | 4 | 4 | 3 | 3 | 5 | 5 | 4 | 5 | | 5 | 4 | | 3 | 3 |
| 2 | 4 | 4 | 0 | 0 | 4 | 4 | 4 | 4 | | 0 | 0 | | 0 | 0 |
| 3 | 5 | 5 | 4 | 4 | 5 | 5 | 4 | 5 | | 4 | 5 | | 3 | 3 |
| 4 | 5 | 4 | 4 | 4 | 5 | 4 | 4 | 4 | | 5 | 4 | | 4 | 4 |
| 5 | 5 | 5 | 4 | 3 | 4 | 5 | 4 | 4 | | 4 | 5 | | 4 | 4 |
| 6 | 4 | 4 | 4 | 4 | 5 | 4 | 5 | 4 | | 4 | 4 | | 3 | 2 |
| 7 | 5 | 5 | 4 | 4 | 4 | 5 | 5 | 5 | | 5 | 5 | | 4 | 4 |
| 8 | 5 | 4 | 4 | 4 | 5 | 5 | 4 | 5 | | 5 | 4 | | 3 | 3 |
| 9 | 4 | 5 | 4 | 3 | 4 | 5 | 5 | 4 | | 4 | 5 | | 3 | 4 |
| 10 | 4 | 4 | 5 | 5 | 5 | 5 | 5 | 4 | | 5 | 4 | | 4 | 3 |
| 11 | 5 | 5 | 4 | 4 | 5 | 5 | 4 | 4 | | 5 | 4 | | 2 | 3 |
| 12 | 4 | 4 | 4 | 4 | 5 | 4 | 4 | 5 | | 5 | 5 | | 4 | 4 |
| 13 | 5 | 5 | 4 | 4 | 5 | 5 | 5 | 5 | | 5 | 5 | | 4 | 5 |
| 14 | 5 | 5 | 5 | 4 | 5 | 5 | 5 | 5 | | 4 | 4 | | 5 | 4 |
| 15 | 5 | 5 | 3 | 3 | 4 | 5 | 5 | 4 | | 5 | 5 | | 3 | 3 |
| 16 | 4 | 4 | 3 | 3 | 4 | 4 | 4 | 4 | | 5 | 5 | | 5 | 5 |

ST6. Round 3: Partial results of expert voting

| Expert Number | Alprazolam tablets  Excitement symptoms Suppress symptoms Psychotic symptoms | | | Eszopiclone tablets  Excitement symptoms Suppress symptoms Psychotic symptoms | | |
| --- | --- | --- | --- | --- | --- | --- |
| 1 | Y | N | Y | Y | Y | Y |
| 2 | Y | N | Y | N | N | N |
| 3 | Y | N | N | Y | N | N |
| 4 | Y | N | N | Y | N | N |
| 5 | Y | N | N | Y | Y | Y |
| 6 | Y | N | N | Y | N | N |
| 7 | Y | N | N | Y | N | N |
| 8 | Y | N | N | Y | N | N |
| 9 | Y | N | N | Y | N | N |
| 10 | Y | N | N | Y | N | N |
| 11 | Y | N | N | Y | N | N |
| 12 | Y | N | Y | Y | N | N |
| 13 | Y | N | N | Y | N | N |
| 14 | Y | N | N | Y | N | N |
| 15 | Y | N | Y | Y | N | N |
| 16 | Y | N | N | Y | Y | Y |

Y: agreed for pilots, N: disagreed for pilots.
