# Supplementary material for: Consensus on the pharmacological treatment of acute stress disorder in Chinese pilots: a Delphi study
Source: BMC Psychiatry. 2023 Sep 8;23:664. doi: 10.1186/s12888-023-05145-5 (PMC10492406; doi:10.1186/s12888-023-05145-5)
Supplement: Supplementary file 4 — ST4. Round1: Partial results of expert voting; ST5. Round2: Partial results of expert voting [file 12888_2023_5145_MOESM4_ESM.docx]

| Expert familiarity status | Quantification values |
| --- | --- |
| More familiar | 1 |
| Familiar | 0.8 |
| General | 0.6 |
| Unfamiliar | 0.4 |
| Ignorant | 0.2 |

ST2. Expert familiarity self-assessment form
